# Functional neuroimaging of visuo-vestibular interaction

**DOI:** 10.1007/s00429-016-1344-4

**Published:** 2016-12-10

**Authors:** R. E. Roberts, H. Ahmad, Q. Arshad, M. Patel, D. Dima, R. Leech, B. M. Seemungal, D. J. Sharp, A. M. Bronstein

**Affiliations:** 1 Neuro-otology Unit, Division of Brain Sciences, Charing Cross Hospital, Imperial College London, London, UK; 2 Computational, Cognitive and Clinical Neuroimaging Laboratory, Division of Brain Sciences, Imperial College London, Hammersmith Hospital, London, UK; 3 Department of Psychology, City, University of London, London, UK; 4 Department of Neuroimaging, Institute of Psychiatry, Psychology and Neuroscience, King’s College London, London, UK

**Keywords:** Visual–vestibular interaction, Visual motion, Caloric, Vestibular, Visual dependency

## Abstract

The brain combines visual, vestibular and proprioceptive information to distinguish between self- and world motion. Often these signals are complementary and indicate that the individual is moving or stationary with respect to the surroundings. However, conflicting visual motion and vestibular cues can lead to ambiguous or false sensations of motion. In this study, we used functional magnetic resonance imaging to explore human brain activation when visual and vestibular cues were either complementary or in conflict. We combined a horizontally moving optokinetic stimulus with caloric irrigation of the right ear to produce conditions where the vestibular activation and visual motion indicated the same (congruent) or opposite directions of self-motion (incongruent). Visuo-vestibular conflict was associated with increased activation in a network of brain regions including posterior insular and transverse temporal areas, cerebellar tonsil, cingulate and medial frontal gyri. In the congruent condition, there was increased activation in primary and secondary visual cortex. These findings suggest that when sensory information regarding self-motion is contradictory, there is preferential activation of multisensory vestibular areas to resolve this ambiguity. When cues are congruent, there is a bias towards visual cortical activation. The data support the view that a network of brain areas including the posterior insular cortex may play an important role in integrating and disambiguating visual and vestibular cues.

## Introduction

Visual and vestibular information is critical for allowing the brain to update the position of the body in space, and to distinguish between world- and self-motion. However, occasionally visual and vestibular signals are mismatched, which can lead to false sensations of motion. An example of this is the ‘moving train illusion’, where the movement of a nearby train leads to the feeling of motion in the observer, despite they themselves remaining stationary (von Helmholtz and Southall 2005). The precise mechanism by which the brain combines these multisensory inputs is currently not fully understood.

Evidence from neurophysiological studies in primates suggests that neuronal populations within a network of brain regions respond to visual–vestibular stimuli in subtle but different ways, contingent on the congruency of the combined stimuli. The macaque ventral intraparietal area (VIP) contains approximately equal populations of neurons sensitive to congruent or incongruent stimuli (Chen et al. 2011a), whereas dorsal medial superior temporal area (MSTd) and visual posterior sylvian area (VPS) neuronal populations exhibit stronger preferences for opposing stimuli (Takahashi et al. 2007; Chen et al. 2011b). However, despite these regions activating in response to visual or vestibular stimuli, e.g. (Duffy 1998; Gu et al. 2006; Fetsch et al. 2010), a recent inactivation study in macaques provided causal evidence that MSTd is dominant for visual heading direction thresholds; VPS and parieto-insular vestibular cortex (PIVC) areas are dominant for vestibular signals, but inactivation of VPS had no functional consequences (Chen et al. 2016). This suggests that in the macaque, MSTd and VPS/PIVC regions directly contribute to heading perception in response to either visual or vestibular stimuli.

In humans, the neural mechanisms responsible for integrating visual motion and vestibular cues are less well defined, with brain responses to visual and vestibular stimuli often studied separately. Early investigations into visual motion processing reported increased activity in a network of cortical brain areas including occipito-temporal cortex, posterior parietal cortex and a number of subcortical structures, with reduced activation within posterior insular cortex (Brandt et al. 1998; Dieterich et al. 1998; Bense et al. 2001; Kleinschmidt et al. 2002). Caloric vestibular stimulation has been associated with modulation of activity in a similar network of brain regions, but in the opposite direction, with deactivations in visual motion regions (Bense et al. 2001) and increased activity in posterior insular and somatosensory cortex.

These patterns of activity led to the hypothesis that visual and vestibular inputs interact via a mechanism of ‘reciprocal inhibition’, whereby both systems compete to suppress the other in order to produce a coherent sense of self-motion (Brandt et al. 1998). However, this model was developed based on extrapolations from experiments where only one stimulus was employed, either visual or vestibular. Evidence from more recent investigations has elaborated on this picture and suggests that regions within intraparietal sulcus and cingulate sulcus visual area (CSv) respond preferentially to optic flow stimuli which are consistent with self-motion (Wall and Smith 2008; Cardin and Smith 2009). There is also evidence to suggest that PIVC and posterior insular cortex (PIC) have functionally specific roles, being differentially activated in response to object motion (Frank et al. 2016). Those studies that have combined visual and active vestibular stimulation (e.g. galvanic or caloric stimulation) have reported a range of different patterns of activity changes compared to unimodal stimulation (Deutschländer et al. 2002; Cutfield et al. 2014; Della-Justina et al. 2014; Frank et al. 2014), with suggestive evidence that separate populations of neurons exist which are sensitive to congruent or incongruent stimuli in the roll plane within hMST, and a putative homologue to macaque ventral parietal sulcus (pVIP) (Billington and Smith 2015). However, visual–vestibular mismatch is just one example of a situation where the brain has to resolve conflict between contradictory inputs. There is already an extensive literature on response conflict, where the challenge of resolving conflicting inputs has been most closely associated with a brain network including both the insular cortex, inferior frontal gyrus and medial frontal structures pre-supplementary motor area (pre-SMA) and anterior cingulate cortex (Nachev et al. 2005; Sridharan et al. 2008; Sharp et al. 2010; Roberts and Husain 2015; Kolling et al. 2016). It is possible that these regions may also play a significant role in facilitating the resolution of perceptual conflicts.

On a behavioural level, the relative perceptual weighting afforded to visual or vestibular cues has been shown to vary across the normal population, and is termed ‘visual dependency’ (Witkin et al. 1975). This psychophysical index of sensory integration can be measured using a task (Dichgans et al. 1972) that determines the degree to which an individual’s subjective perception of verticality, the subjective visual vertical (SVV), is influenced by visual stimuli (Guerraz et al. 2001). Therefore, as part of the study we also assessed visual dependency to test whether this metric predicted individual differences in activation within candidate brain regions, as this could relate to individual differences in the complexion of neuronal populations sensitive to visual or vestibular stimuli.

In the current study, we selectively combined visual and vestibular stimuli to generate conditions that indicated either self-motion in the same direction (congruent), or were conflicting (incongruent). This approach has been employed in previous behavioural experiments to investigate concurrent visuo-vestibular stimulation (Probst et al. 1995; Loose et al. 1999). When the slow phase of the vestibular nystagmus was in the same direction as that elicited by the visual motion the condition was *congruent* (as this is what a person rotating in, and gazing at, the real world would experience). Our hypothesis was that incongruent stimuli would be associated with increased activation in brain regions homologous to those in the macaque which also display a high degree of selectivity for incongruent stimuli, such as hMST or VPS/PIVC regions (Takahashi et al. 2007; Chen et al. 2011a).

## Methods

### Participants

Twenty-eight healthy right-handed subjects were recruited (14 male, mean age 23.6, SD 4.9), with no history of otological, neurological or ophthalmological disease. Eighteen took part in the MRI experiment, and ten in the behavioural experiment. The function of the peripheral vestibular organs can be tested by irrigating the inner ear with water above or below body temperature (Fitzgerald and Hallpike 1942). Function is assessed by measuring the characteristic eye movements (nystagmus) evoked by the vestibular ocular reflex (VOR). A cold (30°) caloric irrigation induces a slow phase eye movement in the direction of the irrigated ear; a warm (44°) irrigation induces the opposite effect. A standard clinical caloric irrigation test was performed using both cold and warm water temperatures to confirm healthy vestibular function in all participants and to familiarise them with the sensation. All subjects were right-handed as indexed by the Edinburgh Handedness Inventory (mean score 83%). Data from one participant who did not complete the experiment were excluded. All participants provided written informed consent. The study was approved by the Bromley National Research Ethics Committee.

### Experimental design

#### MRI experiment

We conducted four experimental runs each lasting approximately 3.5 min using a block design. We used two factors: temperature of caloric irrigation of the right ear (cold or warm), and direction of motion (leftwards or rightwards), to give four possible conditions. The conditions were then grouped into situations in which the visual motion was in the same direction as the slow phase of the vestibular nystagmus (congruent: cold + rightwards motion or warm + leftwards motion), or opposite (incongruent: cold + leftwards motion or warm + rightwards motion). This allowed us to control for differences that might arise from the temperature of stimulation, somatosensory stimulation and the direction of nystagmus, which have previously been shown to induce differential brain activation (Dieterich et al. 2003; Naito et al. 2003; Bense et al. 2006). The speed of the visual stimulus and the duration of the vestibular stimulus were fixed. The peak vestibular response derived from the peak slow phase velocity of the eye movements during irrigation was used as a covariate in the subsequent MRI analysis to account for inter-individual differences in vestibular activation. We employed visual and vestibular functional localisers to use as regions of interest in the analysis of the main experiment, “Visual stimuli” and “Vestibular stimuli”.

#### Behavioural experiment

In addition to the main (fMRI) experiment, we also conducted a behavioural experiment in a separate group of participants outside the scanner to determine the effect that the congruence of the stimuli had on perception of self-motion during cold irrigations of the right ear. The participants lay supine on a couch under low lighting conditions, and viewed a computer screen via a mirror—with the same field of view as in the MRI environment. Participants first viewed the visual motion stimulus and were asked to rate their subjective experience of dizziness on a Likert scale, with the intensity of the standard caloric they received as part of the screening process rated as a ‘5’ on the scale. Using the same design as in the MRI experiment, they then received a cold caloric of the right ear, followed by visual stimuli in combinations that were either congruent or incongruent. The participants rated the peak intensity of dizziness during the caloric irrigation using the Likert scale, and then again for the period when the visual stimulus was present. Eye movements were recorded to ensure that fixation was maintained. Finally, the participants were asked to select which of the two conditions, congruent or incongruent, most closely resembled true self-motion in the real world and to what extent, with ‘10’ equivalent to complete realism.

### Visual stimuli

**Fig. 1:**
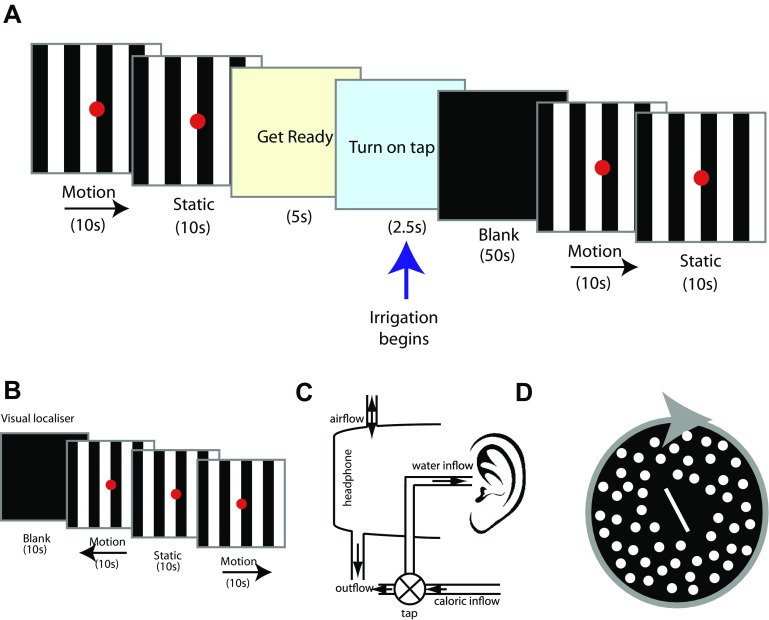
Experimental design and apparatus. **a** Schematic of visuo-vestibular interaction experiment. **b** Schematic of visual motion localiser experiment. **c** Experimental apparatus for irrigating the ear canal inside the MRI scanner. Circulating water was diverted into the ear canal via a manually operated tap, controlled by the participant. The water exits via the outflow pipe and the pressure is equalised by the airflow inlet. **d** Psychophysical stimulus used to measure subjective visual vertical (while background is static) and visual dependency (background rotating in roll plane)

The visual stimuli comprised eight alternating black or white stripes, each subtending an angle of 1.9°, on a screen with a total visual angle of 15°. The stimuli conditions were either stationary, or moving OKS horizontally to left or right at a velocity of 8°/s, superimposed with a red fixation dot subtending 0.5°. At the beginning of each run, there was a 60 s baseline period of visual stimuli with three 10 s periods of static and three periods of visual motion (Fig. 1a), in a counterbalanced order. The irrigation was performed in low lighting conditions with no visual stimulus. Immediately following the irrigation, the visual stimulus resumed for a further 60 s. A visual localiser was used to functionally localise visual cortex and motion sensitive regions (V5/MT). The visual localiser comprised four different visual stimuli: static, leftward motion, rightward motion, and a black screen, which were identical to the baseline stimuli. Each condition was presented for a period of 10 s, six times in total (Fig. 1b). Eye movements were recorded with an infrared MRI compatible eye tracking system (Ober consulting, Poland). Eye movements were recorded. All visual stimuli were generated using C++.

### Vestibular stimuli

To provide vestibular stimulation within the scanner, a modified headset with inlet and outlet tubes was developed, (Fig. 1c). A standard caloric irrigation nozzle was positioned securely within the ear canal using surgical tape, fitted inside adapted headphones. The head was placed at 30° above the horizontal to ensure maximal activation of the horizontal semi-circular canals, and minimise the possibility of magnetic vestibular stimulation induced by the static magnetic field present within the scanner (Roberts et al. 2011). The irrigation nozzle was connected via a thermally insulated tube to a standard clinical caloric irrigation system (CHART VNG; ICS medical) in the control room. Prior to the start of the irrigation, water was continuously circulated within the tubing to maintain a constant temperature. The participants kept their eyes open throughout the experiment so that eye movements could be recorded. They were asked to fixate on the screen dot during the periods of visual stimulation (see “Results”). To initiate each irrigation, the participants were required to turn on a small plastic tap when cued, which was connected via a 10 cm pipe to the circulating water supply. This approach was employed so that the participants were in control from the onset of the irrigation to minimise their discomfort, and any potential head movements, which can occur when caloric irrigation begins. The right ear canal was irrigated for 50 s with 250 ml of ‘cold’ (30 °C) or ‘warm’ (44 °C) sterilised water. Each volunteer received two cold and two warm irrigations in a counterbalanced order, always of the right ear. The participants were cued via the presentation screen with the message “get ready” for 5 s and followed by “turn on tap” for 2.5 s. The participants practiced this procedure prior to data collection. A delay of four volumes was employed between the point where the tap was turned on, and the use of these data for the vestibular localiser to ensure that there was sufficient time for motor activation to subside. During the irrigation, the participants kept their eyes open under low lighting conditions. Although we could have used body temperature caloric stimulation during the baseline period as a control for non-vestibular artefacts of the irrigation, we chose instead to irrigate between the two periods of visual stimuli presentation. Thus, we contrasted baseline visual stimuli with the same visual stimuli after irrigation had ended. Although it is possible that there may have been some non-vestibular activation artefacts, these would have been present during both incongruent and congruent conditions, therefore, effectively cancelled in the main contrast of interest. At the end of each run, participants were asked to rate their subjective experience of dizziness on a Likert scale, rating the intensity of the standard caloric they received as part of the screening process as a ‘5’ on the scale.

### Psychophysical measures: visual dependency

We measured visual dependence immediately prior to the experiment in a quiet testing room, using the Rod and Disk test on a laptop computer as described previously (Cousins et al. 2014), see Fig. 1d. Briefly, subjects were upright and viewed a laptop screen through a viewing cone that excluded extraneous visual orientation cues. The diameter of the cone at the subjects’ eyes was 15 cm with a depth of field of 30 cm, subtending a viewing angle of 39°. The visual stimulus consisted of a white 6 cm rod on a black background. Outside of this central zone, the viewing screen was filled with a 220 off-white dots, each subtending 1.5° of visual field, randomly distributed on a black background. Subjects were instructed to align the rod to their perceived vertical (the subjective visual vertical) under three background conditions: stationary dots; dots rotating clockwise (30°/s); or dots rotating anticlockwise. Subjects performed 15 trials in each condition, with the order of motion conditions randomised across participants. At the beginning of each trial, the rod was set to ±40° from vertical. The rod tilt for each trial was recorded as the difference in degrees between true vertical and the subjects’ final placement of the rod. This provided measures of both subjective visual vertical (SVV) and roll-motion SVV from which could be derived visual dependence, in degrees (=SVV tilt during roll-motion − static SVV tilt). Rod-and-Disk software is available online at: www.imperial.ac.uk/medicine/dizzinessandvertigo.

### Image acquisition

Gradient echo planar MR images were acquired on a Siemens Verio 3T scanner. For each participant, four runs were performed with caloric irrigation (99 volumes) and one run used as a visual localiser (96 volumes). Functional T2*-weighted images were acquired at each of 44 axial, contiguous planes using a gradient echo sequence in an interleaved order (TR 2500 ms, TE 30 ms, flip angle 80°, voxel dimensions 3 × 3 × 3 mm, acquisition matrix 64 × 64). For each participant, a high-resolution T1-weighted anatomical image was acquired in the axial plane for subsequent co-registration (TR 2300 ms, TE 3 ms, TI 900 ms, flip angle = 9°, bandwidth 238 Hz/pixel, voxel dimensions 1 × 1 × 1 mm, matrix size 256 × 192, FOV 240 × 256 mm, slice thickness = 1 mm, number of excitations = 1). Foam padding was used to limit head motion.

### Image processing

#### Processing

For image preprocessing and statistical analysis, we used the SPM8 software package (Wellcome Trust Centre for Neuroimaging, London, UK; http://www.fil.ion.ucl.ac.uk). Images were realigned to correct for movement and normalised into MNI space using each subject’s structural MRI image. The data were then smoothed with an 8 mm Gaussian filter (FWHM).

#### First level analysis

For each participant, the data from the four caloric conditions were concatenated and modelled with a general linear (convolution) model with movement parameters included as confounds. Vectors representing the onset of visual motion, visual static and caloric onsets were convolved with a hemodynamic response function. Additional TRs were then taken to construct the 30 s periods of static or moving visual periods. A high-pass filter (128 s) was employed to remove low-frequency noise, and serial correlations were removed using a first-order auto-regressive model. An explicit mask was used to include only voxels within the brain as part of the analysis. Six movement parameters were included as nuisance covariates. The mean of each session and the transition between the four sessions were also modelled.

#### Visual–vestibular interaction

We focused the analysis primarily on the interaction between visual motion stimuli immediately following the caloric irrigation. Thus, contrast images of brain activations comparing the 30 s of visual motion stimuli during the baseline to the first 30 s of visual motion following the vestibular stimulus were generated.

#### Vestibular localiser

To produce a functional localiser for vestibular activation, we generated contrast images by comparing the final 20 s of the caloric irrigation with the previous 20 s, given that the first 20 s of the irrigation induces virtually no nystagmus. The vestibular response is known to begin to increase after around 30 s, peaking after around 50 s and then maintaining a constant velocity nystagmus for 60 s (Hood and Korres 1979; Guzman-Lopez et al. 2011). This was confirmed by eye movement recordings of vestibular nystagmus during irrigation (see “Results”). This approach controlled for the auditory and somatosensory activation associated with the irrigation, and a 10 s interval was employed following the initiation of the irrigation to exclude any association motor activation.

#### Visual localiser contrasts

To generate a functional localiser for visual cortex, we contrasted brain activation during the moving and static visual stimuli with activity during the blank periods.

#### Second level analysis

Group-level analyses were based on random-effects analyses of the single-subject contrast images using the summary statistic approach. One sample *t* tests were used to investigate the main effect of congruence across the four conditions. Using the vestibular and visual localisers as regions of interest, a statistical threshold of *P* < 0.001 (uncorrected), with a cluster-forming threshold of 20 voxels was used. All results are reported in MNI coordinates.

## Results

### Physiological and subjective measures of vestibular activation

**Fig. 2:**
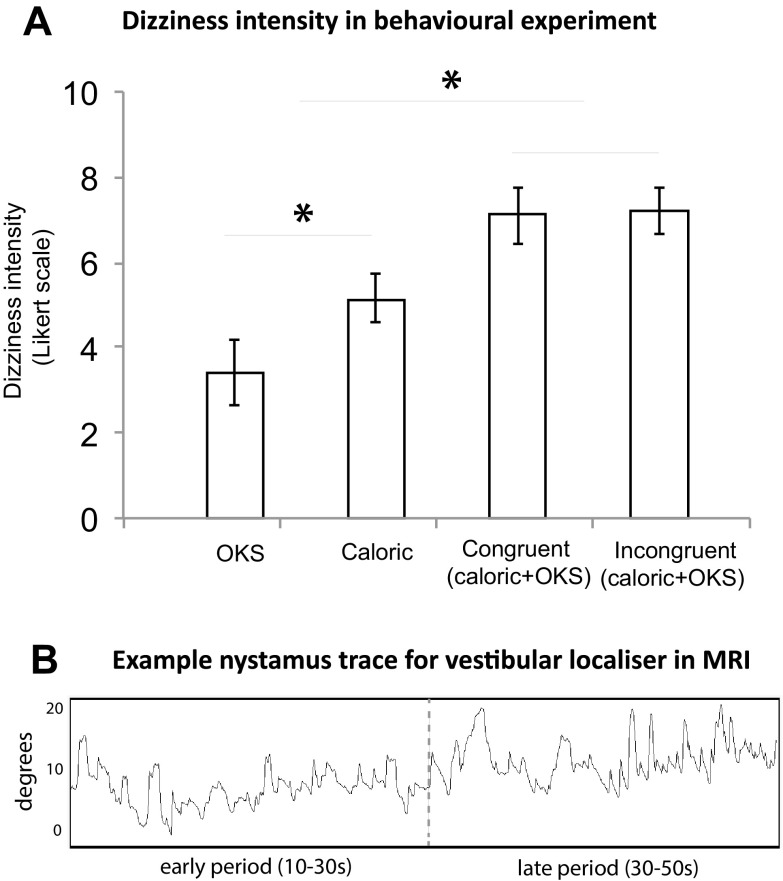
Behavioural measures. **a** Perceived intensity of dizziness in the behavioural experiment for each of the four conditions: optokinetic stimulation (OKS); caloric irrigation of left ear; congruent combination of caloric and OKS; incongruent combination of caloric and OKS. **b** Example trace for nystagmus during early and late irrigation periods for a representative subject

All participants exhibited horizontal vestibular nystagmus, the direction of which was congruent with the expected physiological effect of the irrigation, e.g. right cold irrigation elicited left beating nystagmus (typical example; Fig. 2b). The vestibular nystagmus measurements were taken using eye movements during the final 20 s of each irrigation since this is when nystagmus is manifest (Guzman-Lopez et al. 2011). The mean of the peak slow phase velocity (SPV) for cold irrigations was 14.9°/S, SD 6.92, and for warm irrigations was 13.26°/S, SD 6.37 which occurred in the final period of the irrigation. There was no significant difference between the degree of nystagmus induced by the different temperatures of irrigation (*P* = 0.38, paired samples *t* test), indicating that the intensity of stimulation was consisted across runs. There was a modest trend towards adaptation of the vestibular stimulus when comparing the response during the first irrigation (mean 15.99, SD 7.3) to the fourth irrigation (mean 12.2, SD 6.88) across individuals (*P* = 0.067), which was accounted for by the counterbalanced design. In the first 30 s of irrigation, we did not observe any nystagmus in the participants’ eye movement traces.

To compare the nystagmus present during visual stimuli presentation with fixation, a repeated-measures ANOVA was used with factors TEMP (Cold, Warm) and Direction (Left, Right). There was no significant main effect of temperature *F*(1,16) = 3.61; *P* = 0.69, but there was a significant main effect of direction *F*(1,16) = 74.8, *P* < 0.001. There was no significant interaction (*P* = 0.18). The data demonstrate that the conditions with leftwards OKS had a very small associated leftward nystagmus (NB here a negative slope implies leftward movement): cold left (mean −0.86°/s, SD 0.46); warm left (mean −1.15°/s, SD 0.85). Whereas the rightward OKS conditions demonstrated a rightward nystagmus: cold right (mean 0.94°/s, SD 0.57); warm right (mean 0.98°/s, SD 0.78). We also found similar values for nystagmus during the visual localiser with leftward OKS (mean 0.63°/s, SD 0.3) not significantly different to rightward OKS (mean 0.68°/s, SD 0.47, *P* = 0.56). Thus, during all conditions we observed a small degree of nystagmus with the slow phase in the direction of the OKS.

To ensure that there was consistent fixation throughout the experiment, we measured the variability of eye position by taking the position variance in the *x*-axis during the periods of visual motion stimulation for each of the four conditions. A repeated-measures ANOVA with factors Temp (cold, warm) and direction (left, right) was used. There was no main effect of Temp (*P* = 0.4) or Direction (*P* = 0.76), or interaction (*P* = 0.25). The mean standard deviation for the conditions was: cold leftwards (mean 0.52°, SD 0.23), cold rightwards (mean 0.56°, SD 0.28), warm leftwards (mean 0.51°, SD 0.27), and warm rightwards (mean 0.64°, SD 0.34). The mean deviation across the four conditions was 0.55°, indicating that for 95% of the time eye position was within ±1.1° of the midline. This was comparable with the variability in position values for fixation during the visual localiser (mean 0.51°, SD 0.37).

The mean perceived intensity of dizziness (1–10 Likert scale) was rated with respect to the intensity of the caloric previously experienced outside the scanner, which participants were told was a ‘5’ on the scale. A repeated-measures 2 × 2 ANOVA was used to compare perceived dizziness in response to the vestibular stimulation with factors: Temperature (Cold, Warm) and Direction (Left, Right). There was no significant main effect of TEMP or DIRECTION, or interaction (*P* = 0.15) for any of the four conditions: cold leftwards (mean 4.47, SD 1.78), cold rightwards (mean 4.12, SD 1.63), warm leftwards (mean 4.91, SD 1.80) or warm rightwards (mean 4.88, SD 1.79). There was a weak association between mean subjective intensity and SPV, but this did not reach significance (*r* = 0.36, *P* = 0.15). The adaptation effect on perception was weaker than on the physiological response, with the first irrigation (mean 4.11, SD 1.62) slightly higher than the fourth (mean 4.88, SD 1.79), but not significantly so (*P* = 0.1).

For the visual dependency behavioural task, the mean tilt deviation with a static background, analogous to the subjective visual vertical, was 0.49°, SD 0.35. For the roll-motion conditions, there was no significant difference between line deviation in the presence of clockwise or anticlockwise motion (*P* = 0.17, paired samples *t* test), although the polarity of the deviation was opposite, as expected. Therefore, the mean of these values was taken and then subtracted from the static deviation value. This value was defined as “visual dependency”; the mean value for the group was 2.71°, SD 2.20.

### Behavioural experiment results

In the behavioural experiment, we assessed the intensity and perceptual differences between congruent and incongruent stimuli. When viewing the OKS alone, the average dizziness intensity rating was mean 3.4, SD 2.37 (Fig. 2a). This was significantly lower than during caloric irrigation (mean 5.15, SD 1.75; *P* < 0.02), (*P* < 0.03), paired samples *t* test. The intensity of dizziness experienced during the combined vestibular and visual stimulation was significantly higher than caloric alone in both congruent (mean 7.20, SD 1.75; *P* < 0.001) and incongruent conditions (mean 7.10, SD 2.13, *P* < 0.001). Thus, combined stimulation was perceived as more intense than unimodal, but there was no significant difference between congruent and incongruent conditions (*P* = 0.88). When asked to select the condition that most closely reflected real-world motion, nine out of ten of the participants chose the congruent condition, with a mean realism rating of 4.10, SD 2.23.

### Visual and vestibular localisers

As expected, the vestibular localiser demonstrated significantly increased activation in the later stages of the irrigation (30–50 s) compared to the earlier period (10–30 s) associated with vestibular activation (see Table 1). The primary activations were adjacent to somatosensory association and primary motor cortex, insular cortex, cingulate gyrus and lentiform nucleus, where previous studies have also reported activations, e.g. Suzuki et al. (2001) and Fasold et al. (2002).

**Table 1:** Areas of increased activation following vestibular caloric stimulation

Location	BA	Cluster	Side	*x*	*y*	*z*	Max *t* value	*z* value
Vestibular caloric activation								
Frontal lobe, precentral gyrus	4	61,965	Right	40	−16	50	14.01	6.35
Occipital lobe, inferior occipital gyrus	17	514	Left	−30	−98	−10	6.80	4.60
Anterior lobe, culmen		203	Left	−40	−44	−24	5.41	4.02
Occipital lobe, cuneus	19	354	Left	−18	−88	30	5.23	3.94
Posterior lobe, cerebellar tonsil		28	Right	26	−48	−38	5.04	3.84
Posterior lobe, cerebellar tonsil		51	Right	4	−56	−46	4.94	3.79
Posterior lobe, declive		44	Left	−8	−72	−20	4.44	3.53
Frontal lobe, superior frontal gyrus	9	30	Left	−10	56	36	4.23	3.41
Occipital lobe, lingual gyrus	18	31	Right	10	−74	0	4.19	3.40
Limbic lobe, posterior cingulate	31	23	Right	10	−56	20	4.17	3.38
Parietal lobe, supramarginal gyrus	40	38	Right	50	−52	28	4.02	3.29

Cluster locations are given in MNI coordinates

*BA* Brodmann area

The results of the visual motion localiser using OKS are listed in Table 2. The contrast visual motion > visual static was associated with activations in a number of regions including the associative visual cortex, dorsal posterior cingulate cortex, cerebellar tonsil, middle temporal gyrus and culmen. The contrast visual static > visual motion was associated with increased activation in left and right secondary visual cortex (Fig. 2d). These activations are in line with previous findings where visual motion was investigated with a small-field OKS (Bense et al. 2006; Kikuchi et al. 2009).

**Table 2:** Visual localiser activations for the contrasts visual motion > static, and static > visual motion

Location	BA	Cluster	Side	*x*	*y*	*z*	Max *t* value	*z* value
Contrast: visual motion > static								
Occipital lobe, lingual gyrus	18	7811	R	20	−76	−4	7.96	4.99
Anterior lobe, culmen			R	10	−44	−2	7.57	4.87
Occipital lobe, middle temporal gyrus			R	50	−76	16	7.35	4.79
Occipital lobe, cuneus	18	532	R	4	−76	−30	6.91	4.64
Posterior lobe, declive			L	−10	−74	−20	5.65	4.13
Posterior lobe, inferior semi-lunar lobule			L	−12	−76	−40	5.25	3.95
Frontal lobe, precuneus	31	37	L	−18	−40	42	5.91	4.24
Posterior lobe, cerebellar tonsil			L	−62	−62	−34	5.42	4.03
Temporal lobe, middle temporal gyrus	19	104	L	−42	−80	20	5.39	4.01
Anterior lobe, culmen			L	−44	−48	−30	5.38	4.01
Midbrain, subthalamic nucleus			R	6	−14	4	5.13	3.89
Temporal lobe, middle temporal gyrus	21	74	R	48	4	−26	4.80	3.72
Temporal lobe, superior temporal gyrus	38		R	50	14	−32	4.71	3.68
Sub-lobar, lentiform nucleus			R	28	4	−8	4.43	3.52
Frontal lobe, middle frontal gyrus	10	38	R	26	48	8	4.32	3.46
Parietal lobe, precuneus	7	37	R	10	−42	46	4.27	3.44
Frontal lobe, subcallosal gyrus	47	21	R	22	18	−10	4.25	3.42
Sub-lobar, lentiform nucleus			R	24	14	0	3.87	3.20
Temporal lobe, superior temporal gyrus	22	37	R	64	−54	12	3.94	3.25
Temporal lobe, superior temporal gyrus	22		R	58	−46	16	3.84	3.19

Location	BA	Cluster	Side	*x*	*y*	*z*	Max *t* value	*z* value
Contrast: static > visual motion								
Occipital lobe, lingual gyrus	18	2664	L	−12	−98	−4	13.20	6.22
	18		R	22	−102	−4	9.89	5.53
Occipital lobe, inferior occipital gyrus	18		R	30	−96	−4	9.62	5.46

Cluster locations are given in MNI coordinates

*BA* Brodmann area

Since we were interested in visual and vestibular processing regions, we used the results of the localisers as a mask for the main experiment, described below.

### Interaction between visual motion direction and caloric temperature

**Fig. 3:**
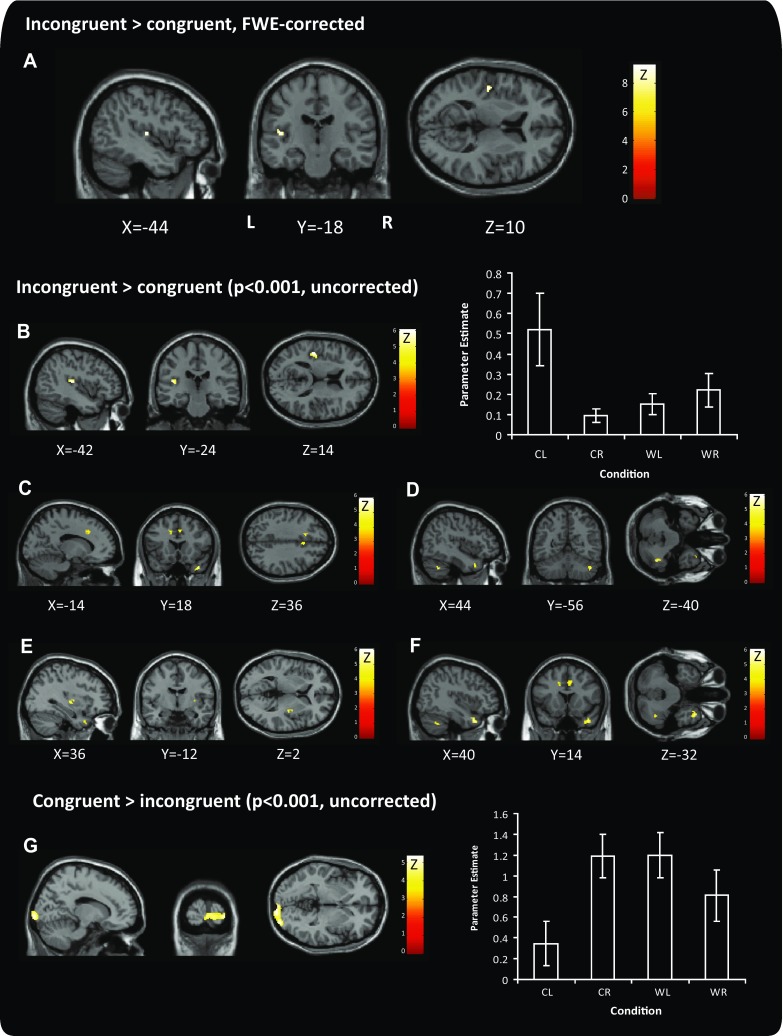
Brain activation in response to congruent or incongruent stimuli combinations. The contrast incongruent > congruent shows the peak activation in **a** posterior insular cortex, at a whole brain corrected level. The reverse contrast showed no significant difference. Restricting the analysis to regions activated using a vestibular or visual stimulus revealed activation in **b** posterior insular/transverse temporal area, with parameter estimates for this region for each condition **c** cingulate gyrus, **d** cerebellar tonsil, **e** claustrum and **f** superior temporal gyrus. **g** The contrast congruence > incongruent was associated with increased activation in primary and secondary visual cortex; parameter estimates are shown on the *right*. All activations are superimposed on a canonical single subject T1 structural image template. All coordinates are in MNI space. *CL* cold leftwards, *CR* cold rightwards, *WL* warm leftwards, *WR* warm rightwards. *Heat bars* indicate *z*-statistic

The central hypothesis of the experiment was that neural activity would be modulated by the congruency of the visual and vestibular motion stimuli. This was shown by the interaction between visual motion (L = left; R = right) and caloric temperature (C = cold; W = warm); see Fig. 3 and Table 3. The contrast incongruent > congruent (CL + WR > CR + WL) revealed a cluster of voxels in the left posterior insular cortex which was significantly activated (Fig. 3a), and was robust to a whole brain FWE correction (*t* = 9.3, *x* = −44, *y* = −18, *z* = 10, *k* = 20), but there were no significant differences for the contrast congruent > incongruent.

**Table 3:** Brain activation in response to congruence contrasts, incongruent > congruent, and congruent > incongruent

Location	BA	Side	Cluster	*x*	*y*	*z*	Max *t* value	*z* value
Contrast: incongruent > congruent (*P* < 0.05, FWE-corrected)								
Posterior insular/transverse temporal gyrus	41	Left	20	−44	−18	10	9.26	5.37
Temporal lobe, transverse temporal gyrus	41	Left	2	−56	−18	10	7.99	5.00

Location	BA	Side	Cluster	*x*	*y*	*z*	Max *t* value	*z* value
Contrast: incongruent > congruent (*P* < 0.001, uncorrected)								
Posterior insular/transverse temporal gyrus	41	Left	58	−42	−24	14	6.05	4.30
Temporal lobe, superior temporal gyrus	38	Right	67	40	14	−32	5.66	4.13
Sub-lobar, claustrum		Right	29	36	−12	2	5.52	4.07
Posterior lobe, cerebellar tonsil		Right	36	44	−56	−40	5.32	3.98
Frontal lobe, medial frontal gyrus	32	Right	54	8	12	42	5.04	3.84
Limbic lobe, cingulate gyrus	32	Left	34	−14	18	36	4.52	3.57

Location	BA	Side	Cluster	*x*	*y*	*z*	Max *t* value	*z* value
Congruent > incongruent (*P* < 0.001, uncorrected)								
Occipital lobe, inferior occipital gyrus	18	Right	416	34	−96	−2	5.44	4.04
Occipital lobe, cuneus	18			16	−102	−2	5.27	3.96
Occipital lobe, cuneus	17			4	−100	−2	5.09	3.87

Cluster locations are given in MNI coordinates

*BA* Brodmann area

To explore the data further, we then used vestibular and visual localisers to constrain the regions of interest. Using this approach, there was increased activity bilaterally in the border between posterior insular and transverse temporal areas, primarily in the left hemisphere. There were also clusters of increased activity within cerebellar tonsil, claustrum, medial frontal and cingulate gyrus and were driven primarily by the strength of the CL condition in this contrast (Fig. 3b–f). The reverse contrast, congruent > incongruent (CL + WR < CR + WL), was associated with activity within occipital regions, including inferior occipital gyrus and cuneus (Fig. 3g), and stronger activation in the congruent CR and WL conditions.

### Main effects: direction of visual motion and caloric temperature

To examine the main effect of direction of visual motion during combined visual and vestibular stimulation, we compared activation during rightward motion to leftward motion in the post-caloric phase. The leftwards > rightwards contrast revealed significantly higher cluster of activation in the posterior lobe of the cerebellum, whereas the rightwards > leftwards was associated with increased activity within occipital cortex, left lingual gyrus and cuneus (Table 4).

**Table 4:** Brain activation in response to main effect of direction of motion

Location	BA	Side	Cluster size	*x*	*y*	*z*	Max *t* value	*z* value
Contrast: leftwards > rightwards								
Posterior lobe, cerebellar tonsil		Right	35	42	−50	−36	4.43	3.53

Location	BA	Side	Cluster size	*x*	*y*	*z*	Max *t* value	*z* value
Contrast: rightwards > leftwards								
Occipital lobe, lingual gyrus	18	Left	233	−28	−102	−8	7.52	4.85
				−20	−106	−6	4.32	3.47

Cluster locations are given in MNI coordinates

*BA* Brodmann area

We also tested for a main effect of caloric temperature in the post-caloric phase by comparing warm calorics to cold. Using this contrast, we observed no overall significant differences in brain activation between warm and cold caloric temperatures.

### Associations between brain activity and behaviour and physiological indices

To investigate the association between brain activity, behavioural indices, and subjective measures of vestibular activation, we included these in the second level analysis as covariates for each condition across subjects. The interaction between peak subjective dizziness and brain activation in the incongruent > congruent contrast revealed a small cluster which was significantly positively correlated with activity in a region within inferior temporal gyrus, BA = 20 (*x* = −50; *y* = −34; *z* = −12; *P* < 0.01 uncorrected, *k* = 20). Our behavioural measures of static SVV and visual dependency were not significantly correlated with activity within this contrast.

## Discussion

A proposed theory of visuo-vestibular interaction is the reciprocal inhibition hypothesis suggested by Brandt et al. (1998). Subsequent studies have found supporting evidence for this framework by examining the patterns of activation and relative deactivation associated with either vestibular or visual motion stimulation (Bucher et al. 1998; Dieterich et al. 1998; Bense et al. 2001; Deutschländer et al. 2002; Kleinschmidt et al. 2002; Smith et al. 2012). Recent work suggests that within brain areas associated primarily with visual or vestibular activation, it is possible to distinguish the activity of separate populations of neurons which are sensitive to different combinations of these stimuli (Billington and Smith 2015; Frank et al. 2016). In the present study, we sought to extend this work by exploring how the degree of compatibility between long duration visual and vestibular stimuli modulates the brain haemodynamic response. Our hypothesis was that conflicting visual and vestibular stimulation would more intensively recruit brain regions associated with multisensory processing than congruent stimuli combinations. We found that under conditions where visual and vestibular stimuli were in conflict there was increased activation within left posterior insular cortex, a brain region recently shown to respond to both vestibular stimulation and object motion (Frank et al. 2016). When restricting this analysis to include only those regions which were activated in response to either the visual or vestibular localisers, this revealed bilateral posterior insular activations in addition to cerebellar tonsil, cingulate and medial frontal gyri. In contrast, when the stimuli were congruent we found increased activation in primary and secondary visual cortex. Although here we focus on multisensory processing of visual and vestibular inputs, the brain regions in which we observe activation have also repeatedly been implicated in mechanisms of sensory and response conflict (Botvinick et al. 2001; Rushworth et al. 2002; Nachev et al. 2005, 2008; Aron 2011; Roberts and Husain 2015). The insular region shows functional heterogeneity for different modalities such as cognitive control (Sridharan et al. 2008) and sensorimotor tasks, but also an area of conjunction within anterior dorsal insular where these sub-functions might be integrated (Kurth et al. 2010). The precise role of the anterior cingulate cortex (ACC) and medial frontal cortex, particularly pre-supplementary motor area (preSMA), is still a matter of debate, e.g. (Kolling et al. 2016; Shenhav et al. 2016). Whether cingulate records task difficulty or the value of exploring alternative choices in a foraging situation remains open, in part because so many studies have reported robust activation within ACC in a variety of situations (Paus 2001). The role of the PreSMA is slightly more defined, and one school of thought suggests that it holds representations of possible responses, and in order to facilitate switching between different response plans (Isoda and Hikosaka 2007; Nachev et al. 2007). In humans, lesions of preSMA impair the ability to switch between different response plans (Rushworth et al. 2002; Roberts and Husain 2015). When we apply these observations to visuo-vestibular processing, it is possible that these brain areas are engaged when there is conflict between sensory stimuli. It is possible that preSMA could represent the mechanism for switching between perceptual states of world and self-motion, whereas the cingulate could potentially signal visuo-vestibular conflict as a perceptual ‘error’ that requires further resources to process. Our behavioural experiments indicate that the congruent combination of stimuli was perceived by the vast majority of participants as most closely reflecting real-world motion, whereas the incongruent stimulus was not reflective of real-world motion but neither was it a perfect cancellation of vestibular and visual stimuli. This indicates that these stimulus combinations also induced significantly different percepts.

These imaging findings suggest a functional dissociation based on the congruency of visual and vestibular stimuli employed. The primary focus of activation in the incongruent condition was within the posterior insular, in the Sylvian fissure. Within this region, two distinct areas have been identified: PIVC and PIC. Although both respond to vestibular stimulation (Frank et al. 2016), visual object motion induces an increase in activity within PIC, whereas activity in PIVC reduces. This partial deactivation is in line with previous reports based on the reciprocal inhibition hypothesis (Brandt et al. 1998). With respect to the present study, the question is which of these regions—PIVC or PIC—is more likely to be the function site of activation? It has been reported that multivoxel pattern analysis could differentiate congruent versus incongruent populations within PIVC, hMST and pVIP, with an overall trend towards increased activation in the ‘nulled’ condition (Billington and Smith 2015). Here, we also report that a region corresponding to the location of PIVC shows increased activation in our incongruent condition. A critical difference with the present study is the duration of vestibular stimulus employed. Here, we used long duration stimuli (both visual and vestibular), which may have affected the overall vestibular response since the horizontal canals system, but not the vertical, has a velocity storage integrator (Grunfeld et al. 2000). This mechanism may effectively act to reduce the effects of visual–vestibular conflict, and could have reduced the sensitivity to detect areas of brain activation that other groups have reported using GVS combined with roll-motion visual stimuli (Billington and Smith 2015).

The type of visual stimulus has also been shown to effect activation within PIVC. Here, we used small-field OKS, which has been reported to elicit a smaller associated deactivation (Brandt et al. 1998; Deutschländer et al. 2002; Kleinschmidt et al. 2002), whereas full field may induce *increased* activation in PIVC (Akbarian et al. 1988). In the present study, the visual stimulus was constant across conditions; therefore, this would not exclude differential involvement of PIVC when combined with a vestibular stimulus. However, a separate consideration is the presence of vection (visually induced self-motion illusions). A number of human neuroimaging studies have reported activation in the sylvian fissure in response to self-motion sensations (Cardin and Smith 2009; Uesaki and Ashida 2015), corresponding to the location of PIC. The behavioural data in our study indicate that participants perceived the same intensity of self-motion, but that this was qualitatively different between congruent and incongruent conditions. Although we conducted the behavioural and MRI experiments in separate groups, the pattern of behaviour was highly consistent across individuals. Therefore, the perception of self-motion per se is unlikely to have provided a differential result in this case, although the degree of similarity with real-world motion may explain the differences between conditions. Given these factors, it is reasonable to conclude that the activation in the incongruent condition is likely to have been activity within the PIVC, but not PIC region.

It is also possible that differences in eye position during fixation could have affected the results. Analysis of eye movements during the visual stimuli showed that the OKS induced a small nystagmus of approximately 1°/s (recall subjects were fixating a central stationary target). However, participants were able to maintain fixation within 1° of the midline for 95% of the acquisition time, and there was no significant difference in variability between conditions. Although the process of fixation is known to be mediated by Purkinje cells in the vestibulo-cerebellum (Keller and Daniels 1975), by requiring participants to fixate across conditions, any activity associated with visual suppression would not be prominent in a contrast analysis in view of our experimental design. Therefore, it is unlikely that this was a significant contributor to the overall findings.

In the congruent condition, we observed increased activity within primary visual cortex. Activity in visual areas has been reported previously, and combined stimuli (visual and vestibular) have been associated with an overall decrease in activation (Deutschländer et al. 2002). It is possible that in the congruent condition the relative gains of the visual and vestibular stimuli led to an overall increase in visual activation that effectively suppressed the vestibular response. However, this scenario would not occur in the incongruent condition where the stimuli were always in opposition. We also observed significant lateralisation in our results. In the main effect analyses, we examined the influence that direction of visual motion (rightwards or leftwards) and temperature of vestibular stimulation (cold or warm) had on brain activity. Leftward motion was associated with a cluster of higher activation in the posterior cerebellum, whereas during rightwards motion there was greater activity within occipital cortex, left lingual gyrus and cuneus. Such asymmetries have been observed in previous studies of visual and proprioceptive integration (Cutfield et al. 2014), including a report that directly tested the difference between leftward and rightward OKS (Bense et al. 2006). This effect was also present in the interactions, with left PIVC active for incongruent stimuli and a right hemisphere bias for the congruent condition. It is possible that this pattern of activation is a result of hemispheric dominance in vestibular processing as suggested by Dieterich et al. (2003), who reported greater activation in the right hemisphere with right-handers for irrigation of the right ear, following from previous work showing evidence of hemispheric lateralisation for visual stimuli (Dieterich et al. 1998). However, the present study differs in that we presented visual stimuli concurrent with vestibular activation. In addition, the caloric localiser in this study demonstrated significant bilateral activation in our group of participants; therefore, it is possible that the Dieterich study did not detect bilateral activity due to the power of the experiment, or that the presence of visual stimuli interacting with the vestibular stimuli engages a network of brain regions which modulate these effects. The lateralisation we observed for the incongruent condition could be a consequence of the unilateral vestibular stimulation we employed, although this is unlikely given the balance of the design. A more probable basis is the particular combination of the side of stimulation and the dominant hemisphere for vestibular processing in these individuals. We have previously reported that modulation of brain activity specifically in the left (but not right) posterior parietal area using tDCS suppresses the vestibular response to caloric irrigation and susceptibility to motion sickness (Arshad et al. 2014, 2015), suggesting the existence of a more complex relationship between vestibular processing and hemispheric lateralisation of function. However, it is worth noting that a limitation of this study is that we irrigated the right ear only; therefore, a comprehensive comparison of the effects of temperature of stimulation would have to compare the effects of stimulating both ears.

A significant consideration for any study investigating the neural correlates of vestibular processing is the effect that MRI has on the peripheral vestibular system. Recent research into magnetic vestibular stimulation (MVS) has demonstrated that vestibular activation occurs in both humans and animals due to the presence of the static magnetic field (Roberts et al. 2011; Ward et al. 2014b), as the Lorentz force generated by the static magnetic field on the cupula is sufficient to induce nystagmus (Antunes et al. 2012). The degree of vestibular activation is now known to be related to the position of the head (Roberts et al. 2011), and the induced nystagmus relies on the interaction between signals from semicircular canals in both ears (Ward et al. 2014a). In the present study, we aimed to minimise the effect of MVS by choosing a head position which has been reported to elicit the lowest levels of MVS (Roberts et al. 2011); however, this does not guarantee that there was no MVS present, although this would have been consistent throughout the experiment and hence cancelled by the experimental design. A second point regarding MVS is the possibility of interaction with laterality effects—which has been reported with respect to vestibular processing (Dieterich et al. 2003). In the presence of MVS that induces nystagmus in a particular direction, this presents a biased picture of neural processing. As we only irrigated one ear, it is possible that the MVS bias might interact differently with visuo-vestibular processing dependent on the ear being stimulated. Therefore, future studies should consider how to accommodate MVS effects with their experimental design.

We also explored whether the activity in either contrast (congruent or incongruent) predicted individual differences in subjective measures of vestibular activation, or psychophysical behavioural indices. This revealed a positive association between subjective dizziness and a small region within inferior temporal gyrus. It is possible that the relationship between subjective dizziness and the inferior temporal gyrus might relate to an increased load on visual processing regions during higher levels of dizziness. However, it is important to note that the correspondence between vestibular perceptual states and physiological measures is frequently heteroskedastic (e.g. Kolev 2002), and in this case subjective dizziness and peak slow phase of vestibular nystagmus were not significantly correlated. Our psychophysical index of sensory integration, visual dependency, did not predict activation levels in either condition, possibly due to the limited range of behavioural variability in healthy individuals, or that visual dependency is likely a function of a range of variables, including psychological factors (Witkin and Asch 1948), which potentially recruits a distributed network of brain regions. We elected not to include the slow phase velocity of eye movements during irrigation to investigate individual differences with behaviour, instead including it within the general linear model as a covariate to account for different levels of vestibular activation at a neural level. Although it could be argued that any difference between vestibular nystagmus and visually induced nystagmus could constitute an incongruent stimulus, our primary aim was to explore differences between maximal and relatively minimal conflict conditions. Including differences in visuo-vestibular matching may provide a more sensitive parametric approach for assessing individual response to the level of congruency between stimuli.

## Conclusion

Here, we examined how the interaction of vestibular and visual processing varies with the relative congruence of the stimuli. The data suggest that under conditions of visuo-vestibular mismatch, there is preferential activation of the PIVC region. This may reflect a function of this area in disambiguating conflicting sensory inputs, and/or a heavier reliance on vestibular cues during periods of sensory ambiguity. These findings could be of potential clinical significance for dizzy patients with increased visual motion sensitivity, such as visually induced dizziness (Guerraz et al. 2001) and persistent postural–perceptual dizziness (PPPD) (Thompson et al. 2015), as reports suggest that greater grey matter volume in insular regions is positively associated with clinical status (Helmchen et al. 2009). This finding supports the possibility that deficits in the capacity to combine or select between competing sensory inputs could underlie prolonged periods of sensory ambiguity leading to dizziness (Cousins et al. 2014). By identifying brain areas responsive to visuo-vestibular mismatch, it may be possible to develop interventions such as focal neuro-modulation to ameliorate symptoms in patient populations.

## References

[CR1] Akbarian S, Berndl K, Grüsser O-J (1988). Responses of single neurons in the parietoinsular vestibular cortex of primatesa. Ann N Y Acad Sci.

[CR2] Antunes A, Glover PM, Li Y (2012). Magnetic field effects on the vestibular system: calculation of the pressure on the cupula due to ionic current-induced Lorentz force. Phys Med Biol.

[CR3] Aron AR (2011). From reactive to proactive and selective control: developing a richer model for stopping inappropriate responses. Biol Psychiatry.

[CR4] Arshad Q, Nigmatullina Y, Roberts RE (2014). Left Cathodal Trans-Cranial Direct Current Stimulation of the Parietal Cortex Leads to an Asymmetrical Modulation of the Vestibular-Ocular Reflex. Brain stimul.

[CR5] Arshad Q, Cerchiai N, Goga U (2015). Electrocortical therapy for motion sickness. Neurology.

[CR6] Bense S, Stephan T, Yousry TA (2001). Multisensory cortical signal increases and decreases during vestibular galvanic stimulation (fMRI). J Neurophysiol.

[CR7] Bense S, Janusch B, Schlindwein P (2006). Direction-dependent visual cortex activation during horizontal optokinetic stimulation (fMRI study). Hum Brain Mapp.

[CR8] Billington J, Smith AT (2015). Neural mechanisms for discounting head-roll-induced retinal motion. J Neurosci.

[CR9] Botvinick MMM, Braver TTS, Barch DDM (2001). Conflict monitoring and cognitive control. Psychol Rev.

[CR10] Brandt T, Bartenstein P, Janek A, Dieterich M (1998). Reciprocal inhibitory visual–vestibular interaction. Visual motion stimulation deactivates the parieto-insular vestibular cortex. Brain.

[CR11] Bucher SF, Dieterich M, Wiesmann M (1998). Cerebral functional magnetic resonance imaging of vestibular, auditory, and nociceptive areas during galvanic stimulation. Ann Neurol.

[CR12] Cardin V, Smith AT (2009) Sensitivity of human visual and vestibular cortical regions to egomotion-compatible visual stimulation. Cerebral Cortex bhp268 10.1093/cercor/bhp268 PMC2901022 20034998

[CR13] Chen A, DeAngelis GC, Angelaki DE (2011). Representation of vestibular and visual cues to self-motion in ventral intraparietal cortex. J Neurosci.

[CR14] Chen A, DeAngelis GC, Angelaki DE (2011). Convergence of vestibular and visual self-motion signals in an area of the posterior sylvian fissure. J Neurosci.

[CR15] Chen A, Gu Y, Liu S (2016). Evidence for a causal contribution of macaque vestibular, but not intraparietal, cortex to heading perception. J Neurosci.

[CR16] Cousins S, Cutfield NJ, Kaski D (2014). Visual dependency and dizziness after vestibular neuritis. PLoS One.

[CR17] Cutfield NJ, Scott G, Waldman AD et al (2014) Visual and proprioceptive interaction in patients with bilateral vestibular loss. NeuroImage Clin 4:274–282 10.1016/j.nicl.2013.12.013 PMC4107374 25061564

[CR18] Della-Justina HM, Gamba HR, Lukasova K et al (2014) Interaction of brain areas of visual and vestibular simultaneous activity with fMRI. Exp Brain Res 1–16 10.1007/s00221-014-4107-6 25300959

[CR19] Deutschländer A, Bense S, Stephan T (2002). Sensory system interactions during simultaneous vestibular and visual stimulation in PET. Hum Brain Mapp.

[CR20] Dichgans J, Held R, Young LR, Brandt T (1972). Moving visual scenes influence the apparent direction of gravity. Science.

[CR21] Dieterich M, Bucher SF, Seelos KC, Brandt T (1998). Horizontal or vertical optokinetic stimulation activates visual motion-sensitive, ocular motor and vestibular cortex areas with right hemispheric dominance. An fMRI study. Brain.

[CR22] Dieterich M, Bense S, Lutz S (2003). Dominance for vestibular cortical function in the non-dominant hemisphere. Cereb Cortex.

[CR23] Duffy CJ (1998). MST neurons respond to optic flow and translational movement. J Neurophysiol.

[CR24] Fasold O, von Brevern M, Kuhberg M (2002). Human vestibular cortex as identified with caloric stimulation in functional magnetic resonance imaging. Neuroimage.

[CR25] Fetsch CR, DeAngelis GC, Angelaki DE (2010). Visual–vestibular cue integration for heading perception: applications of optimal cue integration theory. Eur J Neurosci.

[CR26] Fitzgerald G, Hallpike CS (1942). Studies in human vestibular function: I. Observations on the directional preponderance (“Nystagmusbereitschaft”) of caloric nystagmus resulting from cerebral lesions. Brain.

[CR27] Frank SM, Baumann O, Mattingley JB, Greenlee MW (2014). Vestibular and visual responses in human posterior insular cortex. J Neurophysiol.

[CR28] Frank SM, Wirth AM, Greenlee MW (2016). Visual–vestibular processing in the human sylvian fissure. J Neurophysiol.

[CR29] Grunfeld EA, Okada T, J’auregui-Renaud K, Bronstein AM (2000). The effect of habituation and plane of rotation on vestibular perceptual responses. J Vestib Res.

[CR30] Gu Y, Watkins PV, Angelaki DE, DeAngelis GC (2006). Visual and nonvisual contributions to three-dimensional heading selectivity in the medial superior temporal area. J Neurosci.

[CR31] Guerraz M, Yardley L, Bertholon P (2001). Visual vertigo: symptom assessment, spatial orientation and postural control. Brain.

[CR32] Guzman-Lopez J, Buisson Y, Strutton PH, Bronstein AM (2011). Interaction between vestibulo-spinal and corticospinal systems: a combined caloric and transcranial magnetic stimulation study. Exp Brain Res.

[CR33] Helmchen C, Klinkenstein J, Machner B (2009). Structural changes in the human brain following vestibular neuritis indicate central vestibular compensation. Ann N Y Acad Sci.

[CR34] Hood JD, Korres S (1979) Vestibular suppression in peripheral and central vestibular disorders. Brain J Neurol 102:785–804 10.1093/brain/102.4.785 315811

[CR35] Isoda M, Hikosaka O (2007). Switching from automatic to controlled action by monkey medial frontal cortex. Nat Neurosci.

[CR36] Keller EL, Daniels PD (1975). Oculomotor related interaction of vestibular and visual stimulation in vestibular nucleus cells in alert monkey. Exp Neurol.

[CR37] Kikuchi M, Naito Y, Senda M (2009). Cortical activation during optokinetic stimulation—an fMRI study. Acta Otolaryngol.

[CR38] Kleinschmidt A, Thilo KV, Büchel C (2002). Neural correlates of visual-motion perception as object-or self-motion. Neuroimage.

[CR39] Kolev OI (2002). The directions of nystagmus and apparent self-motion evoked by caloric tests and angular accelerations. J Vestib Res.

[CR40] Kolling N, Wittmann MK, Behrens TE (2016). Value, search, persistence and model updating in anterior cingulate cortex. Nat Neurosci.

[CR41] Kurth F, Zilles K, Fox PT (2010). A link between the systems: functional differentiation and integration within the human insula revealed by meta-analysis. Brain Struct Funct.

[CR42] Loose R, Ayan T, Probst T (1999). Visual motion direction evoked potentials are direction specifically influenced by concurrent vestibular stimulation. Clin Neurophysiol.

[CR43] Nachev P, Rees G, Parton A (2005). Volition and conflict in human medial frontal cortex. Curr Biol.

[CR44] Nachev P, Wydell H, O’Neill K (2007). The role of the pre-supplementary motor area in the control of action. Neuroimage.

[CR45] Nachev P, Kennard C, Husain M (2008). Functional role of the supplementary and pre-supplementary motor areas. Nat Rev Neurosci.

[CR46] Naito Y, Tateya I, Hirano S (2003). Cortical correlates of vestibulo-ocular reflex modulation: a PET study. Brain.

[CR47] Paus T (2001). Primate anterior cingulate cortex: where motor control, drive and cognition interface. Nat Rev Neurosci.

[CR48] Probst T, Loose R, Niedeggen M, Wist ER (1995). Processing of visual motion direction in the fronto-parallel plane in the stationary or moving observer. Behav Brain Res.

[CR49] Roberts RE, Husain M (2015). A dissociation between stopping and switching actions following a lesion of the pre-supplementary motor area. Cortex.

[CR50] Roberts DC, Marcelli V, Gillen JS (2011). MRI magnetic field stimulates rotational sensors of the brain. Curr Biol.

[CR51] Rushworth MFS, Hadland KA, Paus T, Sipila PK (2002). Role of the human medial frontal cortex in task switching: a combined fMRI and TMS study. J Neurophysiol.

[CR52] Sharp DJ, Bonnelle V, De Boissezon X (2010). Distinct frontal systems for response inhibition, attentional capture, and error processing. Proc Nat Acad Sci USA.

[CR53] Shenhav A, Cohen JD, Botvinick MM (2016). Dorsal anterior cingulate cortex and the value of control. Nat Neurosci.

[CR54] Smith AT, Wall MB, Thilo KV (2012). Vestibular inputs to human motion-sensitive visual cortex. Cereb Cortex.

[CR55] Sridharan D, Levitin DJ, Menon V (2008). A critical role for the right fronto-insular cortex in switching between central-executive and default-mode networks. Proc Natl Acad Sci.

[CR56] Suzuki M, Kitano H, Ito R (2001). Cortical and subcortical vestibular response to caloric stimulation detected by functional magnetic resonance imaging. Cogn Brain Res.

[CR57] Takahashi K, Gu Y, May PJ (2007). Multimodal coding of three-dimensional rotation and translation in area MSTd: comparison of visual and vestibular selectivity. J Neurosci.

[CR58] Thompson KJ, Goetting JC, Staab JP, Shepard NT (2015). Retrospective review and telephone follow-up to evaluate a physical therapy protocol for treating persistent postural-perceptual dizziness: a pilot study. J Vestib Res.

[CR59] Uesaki M, Ashida H (2015). Optic-flow selective cortical sensory regions associated with self-reported states of vection. Front Psychol.

[CR60] von Helmholtz H, Southall JPC (2005) Treatise on physiological optics. Courier Corporation

[CR61] Wall MB, Smith AT (2008). The representation of egomotion in the human brain. Curr Biol.

[CR62] Ward BK, Roberts DC, Della Santina CC (2014). Magnetic vestibular stimulation in subjects with unilateral labyrinthine disorders. Front Neurol.

[CR63] Ward BK, Tan GX, Roberts DC (2014). Strong static magnetic fields elicit swimming behaviors consistent with direct vestibular stimulation in adult zebrafish. PLoS One.

[CR64] Witkin HA, Asch SE (1948). Studies in space orientation. IV. Further experiments on perception of the upright with displaced visual fields. J Exp Psychol.

[CR65] Witkin HA, Moore CA, Goodenough DR, Cox PW (1975). Field-dependent and field-independent cognitive styles and their educational implications. ETS Res Bull Ser.

